# Olfactory Marker Protein Expression Is an Indicator of Olfactory Receptor-Associated Events in Non-Olfactory Tissues

**DOI:** 10.1371/journal.pone.0116097

**Published:** 2015-01-30

**Authors:** NaNa Kang, Hyerin Kim, YoonGyu Jae, NaHye Lee, Cheol Ryong Ku, Frank Margolis, Eun Jig Lee, Young Yil Bahk, Min-Soo Kim, JaeHyung Koo

**Affiliations:** 1 Department of Brain Science, DGIST, Daegu, Korea; 2 Department of Information and Communication Engineering, DGIST, Daegu, Korea; 3 College of Medicine, Yonsei University, Seoul, Korea; 4 Department of Anatomy and Neurobiology, University of Maryland School of Medicine, Baltimore, United States of America; 5 Department of Biotechnology, Konkuk University, Chungju, Korea; Mayo Clinic College of Medicine, UNITED STATES

## Abstract

Olfactory receptor (OR)-associated events are mediated by well-conserved components in the olfactory epithelium, including olfactory G-protein (G_olf_), adenylate cyclase III (ACIII), and olfactory marker protein (OMP). The expression of ORs has recently been observed in non-olfactory tissues where they are involved in monitoring extracellular chemical cues. The large number of OR genes and their sequence similarities illustrate the need to find an effective and simple way to detect non-olfactory OR-associated events. In addition, expression profiles and physiological functions of ORs in non-olfactory tissues are largely unknown. To overcome limitations associated with using OR as a target protein, this study used OMP with G_olf_ and ACIII as targets to screen for potential OR-mediated sensing systems in non-olfactory tissues. Here, we show using western blotting, real-time PCR, and single as well as double immunoassays that ORs and OR-associated proteins are co-expressed in diverse tissues. The results of immunohistochemical analyses showed OMP (+) cells in mouse heart and in the following cells using the corresponding marker proteins c-kit, keratin 14, calcitonin, and GFAP in mouse tissues: interstitial cells of Cajal of the bladder, medullary thymic epithelial cells of the thymus, parafollicular cells of the thyroid, and Leydig cells of the testis. The expression of ORs in OMP (+) tissues was analyzed using a refined microarray analysis and validated with RT-PCR and real-time PCR. Three ORs (olfr544, olfr558, and olfr1386) were expressed in the OMP (+) cells of the bladder and thyroid as shown using a co-immunostaining method. Together, these results suggest that OMP is involved in the OR-mediated signal transduction cascade with olfactory canonical signaling components between the nervous and endocrine systems. The results further demonstrate that OMP immunohistochemical analysis is a useful tool for identifying expression of ORs, suggesting OMP expression is an indicator of potential OR-mediated chemoreception in non-olfactory systems.

## Introduction

Chemoreception is an ancient and evolutionarily pivotal physiological system that deciphers both the identity and intensity of distinct environmental stimuli. This system has evolved to maximize the sensitivity and discriminatory capabilities of an organism. Abundant evidence implicates chemoreception as playing a role in the diverse physiological processes of kin recognition and mating [1], pheromone detection [2], mother-infant bonding [3], food preferences [4], central nervous system physiology [5], and even longevity [6]. Olfactory receptor neurons (ORNs) are specialized cells that can transform the detection of a wide range of odor molecules in the external chemical environment to action potentials, which send signals to the olfactory bulb of the brain. Most ORNs express a single olfactory receptor (OR) gene and most ORNs expressing the same gene converge in a single glomerulus (or pair of glomeruli in mammals) [7–11]. In general, olfactory chemoreception depends on a large multigene family of olfactory specific G-protein coupled receptors (GPCRs) that were initially identified in the rat and are now referred to as the OR family [12]. Today, approximately 400 OR genes in human and more than 1000 genes in rodents have been identified, with extensive sequence diversity within their transmembrane domains (homology of 40–90%) [12–14].

The binding of specific ligands (odorant molecules) to GPCRs in membranes of ORNs initiates an olfactory signal transduction cascade through the subunit of the olfactory G-protein (G_olf_)-adenylate cyclase III (ACIII)-cyclic nucleotide-gated (CNG) cation channels and produces nerve impulses, which are sent to the brain where odor perception is processed [15,16]. In addition, multiple molecular steps are involved in odor adaptation, including Ca^2+^-dependent attenuation of ACIII and Ca^2+^ modulation of the CNG ion channels [17]. Among several molecules within the olfactory signaling transduction pathway, it is generally accepted that ORs, G_olf_, ACIII, and olfactory marker protein (OMP) show highly selective expression in the olfactory system [18]. Ca^2+^ accumulation and removal is very important for not only olfactory sensitivity but also the rates of activation, termination, and adaptation of the olfactory signaling pathways. Thus, proper Ca^2+^ regulation is critical for sensing olfactory stimuli [19,20]. OMP, an abundant, small, cytoplasmic protein with expression highly restricted to mature chemosensory neurons in the main olfactory epithelium (OE), the vomeronasal organ, the septal organ, and the Grueneberg ganglion [21,22] is reportedly involved in clearing the elevated Ca^2+^ that follows olfactory transduction [23,24]. Several reports indicate that OMP is indeed a critical participant in modulating olfactory signal transduction [25,26]. However, Reisert *et al*. [27] suggest that OMP participates in a very early step of the olfactory signal transduction cascade.

In recent years, it has become clear that ORs are expressed in many tissues that are not considered classical chemosensory tissues; however, in the majority of these cases, the physiological functions of the ORs remain elusive [28,29]. The non-olfactory (ectopic) expression of ORs was identified by the amplification of the OR gene through PCR-based cloning, as shown in Table 1 of our previous review article [28]. These ORs can be classified into two major groups: one is involved in hormone secretion, such as the secretion of serotonin and renin by external chemical stimuli [30–32], and the other group is responsible for the migration of multiple cell types, such as sperm [33,34], as well as for muscle cell adhesion [35], cell proliferation [36], and cytokinesis [37]. Recently, microarray and RNA-Seq expression analyses have also shown that principal olfactory signaling components (ORs, G_olf_, ACIII, and OMP) are expressed in many non-olfactory tissues [29,38,39]. Despite several reports regarding potential functions and physiology of ectopically expressed ORs [28], caution should be exercised in interpreting the functionality of this OR expression, which is based only on transcript information. However, along with this RNA-based information, identifying OR and OR-associated proteins expressed in specific cell types within selected chemosensory and non-chemosensory tissues may clarify the physiological function of ORs in olfactory as well as non-olfactory systems. It is currently unknown whether subsets of ORs play different physiological and functional roles in different tissues. Thus, in this study, we systematically examined the expression of ORs and OR-associated proteins in non-olfactory tissues using immunohistochemical (IHC) detection of OMP, and screened for potential sites where ORs may function using the protein expression profiles of conserved canonical olfactory signaling components, including G_olf_, ACIII, and OMP.

**Table 1:** Olfactory receptor expression levels in olfactory marker protein-positive non-olfactory tissues using RT-qPCR analyses.

Tissue	Symbol	Synonym	Accession no.	Expression level*
Olfactory bulb	olfr39	MOR144–1	NM_146825	47.22 ± 7.55
	olfr78	MOR18–2, PSGR, MOL2.3	NM_001168503	1146.43± 133.00
	olfr181	MOR184–4	NM_146999	147.65 ± 36.28
	olfr288	MOR286–3P, C85333	NM_001011733	124.30 ± 21.65
	olfr325	MOR275–5	NM_207153.2	761.79 ± 284.86
	olfr378	MOR135–2	NM_147024.2	108.11 ± 21.97
	olfr544	MOR42–3, S6, S79	NM_020289	693.84 ± 74.40
	olfr558	MOR18–1, POGR	NM_147093	583.88 ± 31.10
	olfr895	MOR170–1	NM_146875	158.42 ± 20.52
	olfr1386	MOR256–50	NM_001011741	434.88 ± 93.40
	olfr1392	MOR256–25	NM_146470	135.15 ± 28.28
Bladder	olfr181	MOR184–4	NM_146999	28.72 ± 3.78
	olfr544	MOR42–3, S6, S79	NM_020289	111.95 ± 22.80
	olfr558	MOR18–1, POGR	NM_147093	376.42 ± 29.39
	olfr895	MOR170–1	NM_146875	213.17 ± 44.43
	olfr1392	MOR256–25	NM_146470	59.19 ± 10.79
Thymus	olfr39	MOR144–1	NM_146825	12.51 ± 1.87
	olfr181	MOR184–4	NM_146999	68.02 ± 11.68
	olfr325	MOR275–5	NM_207153.2	176.20 ± 44.83
	olfr378	MOR135–2	NM_147024.2	4.16 ± 0.68
Thyroid	olfr78	MOR18–2, PSGR, MOL2.3	NM_001168503	2942.66 ± 321.93
	olfr181	MOR184–4	NM_146999	14.86 ± 2.98
	olfr288	MOR286–3P	NM_001011733.2	3.14 ± 0.87
	olfr558	MOR18–1, POGR	NM_147093	1322.75 ± 47.32
	olfr544	MOR42–3, S6, S79	NM_020289	677.44 ± 85.04
	olfr1386	MOR256–50	NM_001011741	4.37 ± 0.72
	olfr1392	MOR256–25	NM_146470	38.98 ± 5.12

*Values are mean fM of mRNA ± SEM. Quantitative real-time RT-PCR was used to analyze the levels of mRNA of 11 olfactory receptors (ORs) in mouse tissues. Olfactory bulb was used as a positive control for OR expression.

*OR mRNA was quantified in reference to the *p*CI-rho-olfr544 plasmid standard and normalized to eEF-2 RNA, n = 3 independent mice.

## Materials and Methods

### Total RNA isolation and RT-PCR analyses

All animal procedures were approved by the Daegu Gyeongbuk Institute of Science and Technology’s Institutional Animal Care and Use Committee (DGIST-IACUC_0001). Total RNA from the targeted tissues of C57BL/6 (male, 7 weeks old) mice was extracted using a MagNA Lyser instrument (Roche Molecular Diagnostics GmbH, Penzburg, Germany) with Trizol reagent (Invitrogen, Carlsbad, CA, USA). Briefly, tissue was homogenized in 1 ml of Trizol, and 200 μl of chloroform (Sigma Aldrich, St Louis, MO, USA) was added. Samples were mixed thoroughly and centrifuged at 13,000 × g for 15 min. The upper aqueous phase was transferred, and the RNA was precipitated by the addition of isopropanol (Sigma Aldrich), followed by centrifugation at 13,000 x g for 10 min. The precipitate was washed with ice-cold 70% ethanol (Sigma Aldrich), and the final pellet was suspended in RNase-free water. Further cleaning to eliminate genomic DNA contamination was performed using the RiboClear plus kit (GeneAll, Seoul, Korea). Total RNA (2 μg) was reverse transcribed using a cDNA synthesis kit (Takara Shuzo, Otsu, Japan). For (-) RT, a negative control without reverse transcriptase (RTase), 1 μl of water was added instead of 1 μl PrimeScript RTase. The PCR reaction mixture consisted of the first-strand cDNA template, PCR Master Mix (Takara Shuzo), and primer sets (S1 Table). Each PCR cycle consisted of denaturing at 94°C for 1 min, annealing at a temperature dependent on the calculated melting temperature (Tm) of each OR or for β-actin at 60°C for 1 min, and elongating at 72°C for 1 min with 35 cycles using a T3000 thermocycler (Biometra, Göttingen, Germany).

### Quantitative Real-time PCR analysis

The OR mRNA was quantitated using real-time PCR with Takara SYBR premix (SYBR Premix Ex Taq II, RR820A, Takara Shuzo) according to the manufacturer’s instructions. Reaction mixtures with a final volume of 20 μl consisted of 2 μl of reverse transcribed cDNA, primers, and 2× SYBR Green. The eukaryotic elongation factor-2 (eEF-2) was used as an internal control [40]. The oligonucleotides used as primer sets in this study are shown in S1 Table. The reaction consisted of the following steps: initial denaturing at 95°C for 30 s, then additional 40 cycles of 95°C for 5 s, and annealing at 60°C for 30 s. This was followed by melting curve analyses from 65 to 95°C at every 0.5°C increase in temperature for approximately 5 s. Melting curve analyses confirmed the presence of a single PCR product in each reaction. A real-time PCR instrument (CFX-96 Bio-Rad thermocycler, Bio-Rad Laboratories, Hercules, CA, USA) was used for this experiment. The relative difference in the expression for each sample in individual experiments was determined by normalizing the cycle threshold (Ct) value for each gene against the Ct value of eEF-2, and was calculated with an equation using a standard concentration curve based on a construct of the mammalian expression vector *p*Cl with Rho and the mouse olfr544 (*p*CI:Rho-olfr544). All experiments were conducted three times, and error bars indicate SEM.

### Immunoprecipitation and western blotting

Total tissue extracts were prepared from C57BL/6 mouse tissues in HEPES buffer (20 mM HEPES, 150 mM NaCl, 1 mM EDTA, and 0.5% Triton X-100, pH 7.5) using a MagNA Lyser instrument. The protein concentrations were determined using a standard Bradford protein assay procedure (Bio-Rad). Total protein extracts of each tissue (20 mg, except 3 mg for thyroid and 100 μg for OE) were incubated with 2.5 μl of goat anti-OMP serum (S2 Table) in a final volume of 500 μl at 4°C for 2 h. Protein G-Sepharose 4 Fast Flow beads (20 μl, GE healthcare, Uppsala, Sweden) were added and incubated at 4°C overnight with gentle mixing. The beads were washed with HEPES buffer three times and another three times with phosphate-buffered saline (PBS). The drained beads were then incubated at 95°C in sodium dodecyl sulfate (SDS) sample buffer, electrophoresed using 10–20% SDS—polyacrylamide gel electrophoresis (PAGE), and blotted to nitrocellulose membranes (Whatman, Maidstone, Kent, UK) for western blot analysis. The nitrocellulose membrane was rinsed in TBST (25 mM Tris—HCl, 2.7 mM KCl, 137 mM NaCl, and 0.1% Tween-20, pH 7.4) and blocked for 1 h in 5% nonfat dry milk in TBST. The membrane was incubated overnight at 4°C with rabbit anti-OMP serum (S2 Table), followed by incubation with the secondary antibody, a horseradish peroxidase (HRP)-conjugated donkey anti-rabbit IgG (1:100,000, Jackson ImmunoResearch, Westgrove, PA, USA). In control experiments, antibodies were preincubated with an excess of competing purified soluble OMP protein. Immunoreactive proteins were detected using an enhanced chemiluminescence (ECL, Supersignal-West Pico, Pierce, Rockford, IL, USA) detection system according to the manufacturer’s directions.

### OR constructs and cloning

To clone the full-length sequences of olfr544 into the “Rho-OR” vector, the sequence encoding olfr62 (a gift from Dr. Hiroaki Matsunami at Duke University Medical Center) was excised from its parent vector (*p*CI vector), and the PCR products containing the full-length sequence of olfr544 were ligated to the corresponding sites in this vector. The full-length olfr1386 and olfr1496 sequences were amplified using primers, which added appropriate restriction sites from mouse OE cDNA, and ligated into the corresponding sites in the olfr691 construct, which contained N-terminal Lucy/Flag/Rho tags in a *p*ME18S vector (a gift from Dr. Jennifer L. Pluznick at Johns Hopkins University School of Medicine) [41]. Rho-olfr558 (MOR18–1) and Rho-OR51E1 were purchased from Addgene (Cambridge, MA, USA). All constructed plasmids were fully sequenced before use.

### Immunofluorescence

HEK 293 cells were seeded onto 22 mm coverslips coated with poly-D-lysine and transfected with OR constructs containing accessory proteins RTP1S (a gift from Hiroaki Matsunami), Ric8B, and G_olf_ (a gift from Dr. Bettina Malnic at University of São Paulo, Brazil) to assess universal effects for plasma membrane expression. Briefly, transiently transfected cells were incubated on ice to block endocytosis, washed with 1× PBS, fixed with ice-cold methanol/acetone (1:1, v/v), and then exposed to mouse anti-Rho antibody and rabbit anti-OR antibodies (OR51E1, olfr1496, olfr1386, and olfr544) in PBS with 5% normal horse serum (NHS, Jackson ImmunoResearch) overnight at room temperature as described in S2 Table. For the detection of bound primary antibodies, Cy3-conjugated anti-mouse IgG (1:2000, Jackson ImmunoResearch) and Dylight 488-conjugated anti-rabbit IgG (1:500, Jackson ImmunoResearch) diluted in PBS containing 5% NHS were incubated for 1 h at room temperature in the dark. After washing, the coverslips were inverted and mounted on glass slides with VECTASHIELD (Vector Laboratories, Burlingame, CA, USA). Images were obtained using a biological confocal laser scanning microscope LSM700 (Zeiss, Oberkochen, NY, USA).

### Immunohistochemistry

Mice were anesthetized with 10 mg/kg Zoletil (Virbac, SA, Carros cedex, France) and perfused transcardially with 20 ml of ice-cold PBS followed by 30 ml of 4% freshly prepared phosphate-buffered paraformaldehyde (Sigma Aldrich). The tissue was dissected and postfixed for 2 h in cold fixative and cryoprotected overnight in 30% sucrose at 4°C. Tissues were embedded in OCT compound (Tissue Tek, Sakura Finetek, Torrance, CA, USA) and snap-frozen in dry ice. Cryostat sections (10 μm) of each tissue were attached onto Superfrost-plus microscope slides (Matsunami, Tokyo, Japan), dried at 37°C for 30 min, and stored at-80°C. Tissue sections were treated with 4% NHS and 0.1% Triton X-100 in PBS for 1 h in a humidified chamber to block nonspecific antibody binding. The slides were incubated overnight at 4°C with primary antibodies (shown in S2 Table) diluted in PBS containing 4% NHS and 0.1% Triton X-100. For detection of the bound primary antibodies, Cy3-conjugated anti-goat IgG (1:2,000) and Dylight 488-conjugated anti-rabbit IgG (1:1,000) diluted in PBS containing 0.1% Triton X-100 were incubated for 1 h at room temperature in the dark. After rinsing, the slides were coverslipped with VECTASHIELD containing DAPI (Vector Laboratories, Burlingame, CA, USA), and IHC images were obtained using a confocal laser scanning microscope LSM700. For cell counting, the sections were evaluated with a 20× objective lens in the target region. Some high power images were magnified to the 40× scale for counting. The resolution was at least 1,024 × 1,024 pixels with 12 or 16 bit color. The area of the counted images was approximately 212.35 × 212.35 μm. The thickness of measured confocal Z-stack images was 6–10 μm with 5–10 planes from the Z axis. The cells were counted with comparisons of each Z plane using ZEN software (Zeiss). The counted sections were obtained from three different mice, and two to five different regions were obtained from each mouse. At least nine images were used for statistical analyses.

### New ranking method for OR selection in OMP (+) tissues

Only valid ORs from the Gene Atlas2 microarray data were identified [42,43]. Although a large number of ORs from olfr1 to olfr1570 are mentioned in several studies [14,38,44], only a subset of these has been validated. The following three steps were performed for each OR from number 1 to 1,570 to determine the validity of the ORs: (1) The nucleotide database of the GenBank site (http://www.ncbi.nlm.nih.gov/genbank) was searched for the “olfr<*number*>.” (2) In the search result, the link with an accession number starting with “NM_,” which indicated the mRNA sequence, was selected. (3) The OR was considered valid when the comment section of the result page began with the word “VALIDATED,” and the title of the result page did not include the words “partial” or “pseudo.” A small number (25) of the ORs that were relatively highly expressed in three tissues were selected from the 352 valid ORs.

To limit the number of ORs for further evaluation, *M* was defined as the refined microarray of *m* ORs and *n* tissues (e.g., *m* = 352 and *n* = 78 in the refined microarray), and *M*_*ij*_ was the specific expression of the *i* OR and the *j* tissue, where 1 ≤ *i* ≤ *m* and 1 ≤ *j* ≤ *n*. In addition, π(*M*_*j*_, *M*_*ij*_) was the ranking position of *M*_*ij*_ in the set *M*_*j*_ = {*M*_*1j*_, *M*_2__*j*_, …, *M*_m__*j*_}, and similarly, π(*M*_*i*_, *M*_*ij*_) was the ranking position of *M*_*ij*_ in the set *M*_*i*_ = {*M*_*i*1_, *M*_*i*2_, …, *M*_*i*n_}. The total ranking function τ_j_(i) for a given OR *i* and a given tissue *j* was defined as follows:

$$\tau_{j}(i)\;=\pi (\theta_{j},\pi (M_{j},M_{ij})\;+\pi (M_{i},M_{ij}))$$

where θ_*j*_ = {π(*M*_*j*_, *M*_*1j*_) + π(*M*_*1*_, *M*_*1j*_), π(*M*_*j*_, *M*_*2j*_) + π(*M*_*2*_, *M*_*2j*_), …, π(*M*_*j*_, *M*_*mj*_) + π(*M*_*m*_, *M*_*mj*_)} [14,43].

For example, olfr190 had the sixth highest intensity out of 352 intensities for the thymus, but it had the 58^th^ highest intensity out of 78 intensities for olfr190. The sum of the two rank values is 64, which is the seventh lowest rank value (i.e., highest rank) out of 352 rank values for thymus, and so τ_*thymus*_ (olfr190) equaled 7. The rank positions of the 352 valid ORs for each of the three tissues were evaluated and the top 25 ORs were selected (summarized in S3 Table).

## Results

### OMP expression as an indicator of potential OR-associated events in non-olfactory tissues

The question of whether OMP is expressed with ORs in non-olfactory tissues is interesting because ORs are expressed in many tissues, and OMP is widely accepted as a marker of mature ORNs and associated with an OR signal transduction cascade. In the present study, we first performed conventional RT-PCR analysis to assess the expression of the OMP gene in a broad range of non-olfactory tissues (S1 Fig.). Although the conventional RT-PCR is not quantitative, we detected the OMP genes in each of the 13 tissues investigated, indicating that the OMP as a potential marker of the olfactory signal transduction system may exist in a wide range of non-olfactory tissues and may function as a potential indicator of the OR-associated events in non-chemosensory tissues.

**Fig 1:**
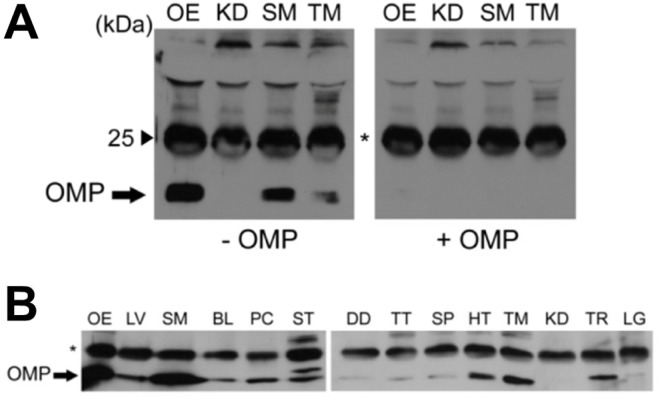
Protein expression profiles of OMP in various mouse tissues. Double immunoassay for olfactory marker protein (OMP). **(A)** In all tissues except for kidney (KD), the results of double immunoassays demonstrate the presence of OMP. Total mouse tissue extract (100 μg for olfactory epithelium [OE] and 20 mg for KD, skeletal muscle [SM], and thymus [TM]) was immunoprecipitated (IP) with goat anti-OMP antibodies and immunoblotted with rabbit anti-OMP antibody (double immunoassay). The apparent molecular mass of OMP (arrow) is 19 kDa in OE, SM, and TM [OMP (-)], and this band is completely blocked by preincubating the OMP antibody with purified recombinant OMP [OMP (+)]. **(B)** Mouse tissue survey for OMP expression. Total tissue extract (100 μg for OE as a positive control, 3 mg for thyroid (TR), and 20 mg for all other tissues) was used for IP. * indicates immunoglobulin. LV, liver; BL, bladder; PC, pancreas; ST, stomach; DD, duodenum; TT, testis; SP, spleen; HT, heart; LG, lung.

We next performed immunological experiments using a double immunoassay technique to support the results of the mRNA studies and determine whether the OMP is expressed at the protein level. As expected, we could not detect the OMP expression with conventional western blot analysis using specific antibodies against the OMP because of the low level of the OMP expression (data not shown). To overcome this challenge, we first tested the specificity of our double immunoassay system. As shown in Fig. 1A, skeletal muscle and thymus except for kidney expressed the OMP [Fig. 1A, OMP (-)]. For this assay, OMP was immunoprecipitated with an anti-OMP antibody from goat conjugated with Protein G-Sepharose using 20 mg of total tissue lysates obtained from each tissue. The isolated immunocomplexes were subjected to western blot analysis with an anti-OMP polyclonal antibody from rabbit. For the positive control, we used the same double immunoassay with 100 μg of tissue lysate obtained from the OE (Fig. 1A, lane OE). The 19 kDa immunoreactive protein band did not appear when tissue was preincubated with the purified recombinant OMP used as a blocking reagent, indicating the specific detection of the OMP in our system [Fig. 1A, OMP (+)]. As shown in Fig. 1B, OMP protein was widely expressed in all but one tested tissue. Skeletal muscle, heart, thymus, and thyroid showed high OMP expression, whereas liver, bladder, pancreas, stomach, duodenum, testis, spleen, and lung were at relatively lower levels. OMP expression was undetectable in the kidney. The endogenous OMP was immunoprecipitated from 20 mg of total tissue lysate obtained from various tissues and from 3 mg of total cell lysate obtained from the thyroid. This OMP protein expression pattern differs from the OMP transcript pattern, in which OMP mRNA is predominantly expressed in the bladder and stomach. In addition, although the OMP protein expression level is low, it was detectable by a double immunoassay, resulting in different expression pattern at various tissues. There are two plausible explanations for low and differential expression of the OMP. The first is that OMP may be broadly expressed at low levels throughout the entire tissue. The second is that OMP may be locally expressed either in a small portion of the tissue or within a specific cell type of each tissue. Nevertheless, OMP expression is only detectable with high resolution methods using whole tissue lysates rather than by conventional methods.

**Fig 2:**
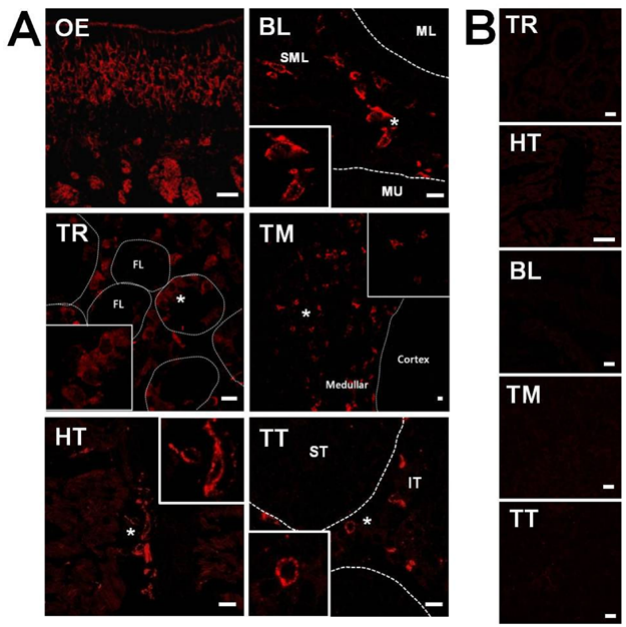
Immunohistochemical detection of OMP in mouse non-olfactory tissues. **(A)** Olfactory marker protein (OMP) staining in olfactory epithelium (OE) was used as a positive control. In the thyroid (TR), OMP (+) cells are observed between follicles (FL) and are similar to a parafollicular cell-like population. OMP (+) cells of the bladder (BL) are assumed to be in the submucosal layer. In the testis (TT), OMP signals are displayed in Leydig cell-like populations belonging to interstitial tissue (IT), and in the thymus (TM), OMP is detected in the medullary area. The asterisks indicate OMP (+) cells. **(B)** Each tissue represents samples stained in the absence of primary antibody. MU, mucosa; SML, submucosal layer; ML, muscle layer; ST, seminiferous tubule. Scale bar: 10 μm.

We next used IHC techniques to discriminate the specific cellular localization of OMP in those tissues showing positive expression. IHC analysis revealed that OMP is significantly detectable within only five non-olfactory tissues, including bladder, thyroid, thymus, heart, and testis (Fig. 2). When the OMP (+) IHC phenotype was examined with the corresponding morphological phenotype in these tissues, we found that the OMP (+) cells in the thyroid are observed between follicles in a parafollicular cell-like population. In the bladder, the OMP (+) cells appeared in submucosal layer. In the testis, the OMP signals were displayed in a Leydig cell-like population within the interstitial tissue and in the thymus, OMP (+) cells were detected in the medullary area. The specific strong immunosignal obtained with goat anti-OMP serum was clearly discriminated from the background autofluorescence (S2A Fig.). In a negative control using testicular sections with normal goat serum, autofluorescence was observed in the peritubular space (S2B Fig.). Together, our data demonstrate that OMP exists in non-olfactory tissues within specific cell types rather than in a regional distribution within small portions of the tissue. These findings provide supporting evidence that OMP may play a more general role in chemosensing in addition to its role in the olfactory system.

### Expression of additional components of OR-associated signaling events in non- olfactory tissues

**Fig 3:**
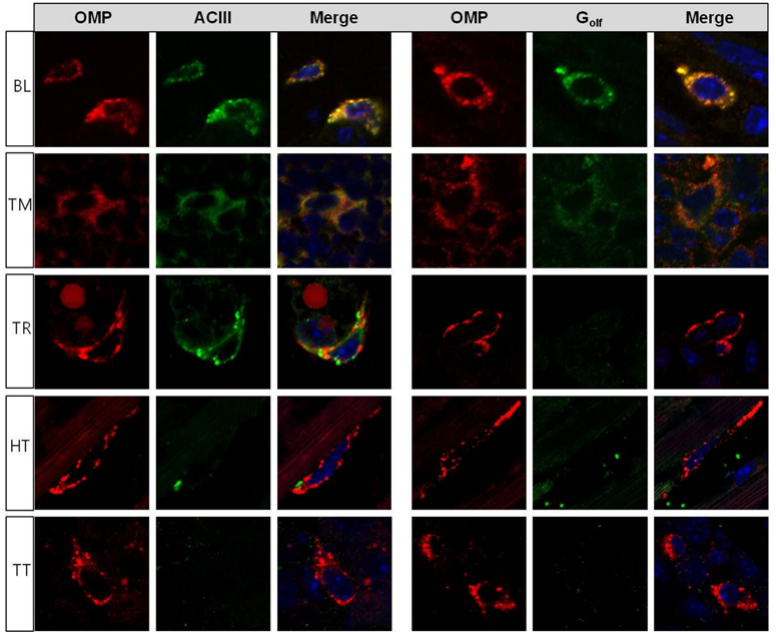
Co-expression of OMP with either ACIII or G_olf_ in non-olfactory tissues using immunohistochemical analysis. The distribution of each olfactory signaling-associated molecule, namely, olfactory marker protein (OMP), adenylate cyclase III (ACIII), and olfactory G-protein (G_olf_), in non-olfactory tissues shows different expression patterns. In bladder (BL) and thymus (TM), OMP (+) cells are co-expressed with ACIII and G_olf_. OMP signals expressed in the thyroid (TR) are colocalized with ACIII, but not with G_olf_. In the heart (HT) and testis (TT), OMP signals are displayed alone, which is not coincident with G_olf_ or ACIII.

Of the several components in the olfactory signal transduction cascade pathway, G_olf_, ACIII, and OMP are co-expressed with ORs. Thus, the simultaneous expression of G_olf_ and ACIII with OMP and the essential OR in a specific cell type within non-olfactory tissues suggests that it is plausible for an OR-associated signaling event to induce an odorant signal. Thus, we surveyed the expression patterns of G_olf_ and ACIII using IHC staining methods with commercially available and highly sensitive antibodies in the five non-olfactory tissues showing positive OMP expression. Robust staining for G_olf_ and ACIII proteins was apparent in cells of the bladder and thymus expressing OMP, indicating that these two tissues might use these essential components (G_olf_, ACIII, and OMP) as chemosensory signaling systems (Fig. 3; BL, bladder, and TM, thymus). Interestingly, in thyroid, immunofluorescence staining was localized to the same cells only for OMP and ACIII and not for G_olf_ (Fig. 3; TR, thyroid). This may be indirect evidence that other G-proteins such as G_q_ or G_i_ or other G_s_ rather than G_olf_ are involved in cell signaling, perhaps even in the olfactory system [45]. However, there were no detectable immunofluorescence signals for G_olf_ and ACIII in the heart or testis, which may be due to the low levels of protein expression, antibody sensitivity, or both (Fig. 3; HT, heart, and TT, testis).

### Identification of OMP (+) cells in bladder, thyroid, and thymus

**Fig 4:**
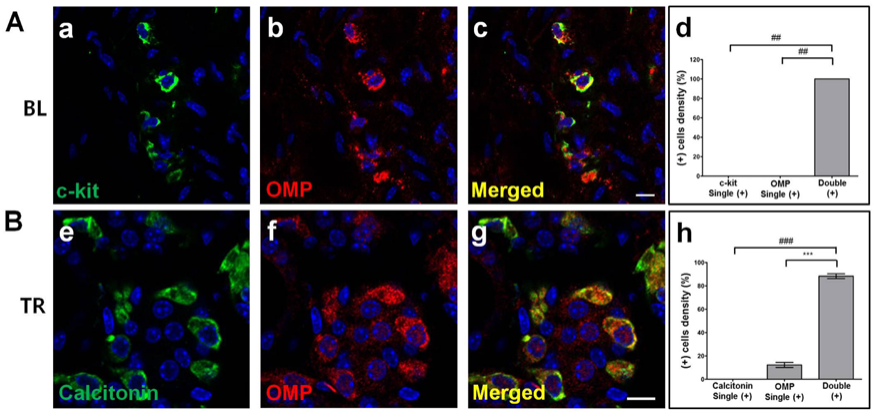
Identification of OMP (+) cells in non-olfactory tissues such as bladder and thyroid. Olfactory marker protein (OMP) expression (red in b and f) is superimposed with each marker using double-labeling immunohistochemical techniques. A representative image is shown for each antibody combination. The left panels show each marker protein with Dylight 488-conjugated anti-rabbit IgG green fluorescence (a, e), the middle panels show OMP with Cy3-conjugated anti-goat IgG red fluorescence (b, f), and the right panels are the merged images of the two individual images in the corresponding rows (c, g). C-kit (green in a), a marker for the bladder (BL in **A**), stains interstitial cells of Cajal (ICC). Calcitonin (green in e) is a marker for parafollicular cells (also known as C cells) of the thyroid (TR in **B**). OMP is expressed in each cell type of mouse tissue as evidenced by colocalization with c-kit (yellow in c) and calcitonin (yellow in g). C-kit/OMP (d) and calcitonin/OMP (h) colocalization was supported by quantitative analysis. ***, *P* < 0.0001 using an unpaired *t*-test; ##, *P* = 0.0004, and ###, *P* = 0.0001 using a Mann—Whitney *U* test OMP + c-kit, or calcitonin versus marker or OMP alone. Results are the means ± SEM of three or four independent areas (n = 3–5 mice). Scale bar: 10 μm.

Given the above demonstration that G_olf_, ACIII, and OMP as the candidates of potential indicator for OR-associated events in non-olfactory tissues were expressed in bladder, thymus, and thyroid, we then identified the immunohistochemically OMP (+) cells in these non-olfactory tissues using IHC double-labeling techniques and quantitative analyses. First, we verified OMP (+) cell populations with cell-specific markers of each tissue from the bladder and the thyroid (Fig. 4A and B).

To study the localization of OMP (+) cells in bladder, mouse bladder tissue sections were subjected to double-immunolabeling and confocal microscopic analysis. We found that OMP (+) cells in bladder were positioned on the lamina propria, having distinctive cell shapes and morphological arrangements in the submucosal layer rather than the muscle and mucosal layers. The OMP (+) cells were mostly distributed submucosally within the interstitial cells of Cajal (ICC) and among the smooth muscle bundles in the lamina propria. The ICC is identified by their c-kit positivity and is thought to mediate signal transmission from neurons to smooth muscles [46–48]. The IHC double-immunolabeling of bladder sections indicated that all OMP (+) cells were positive for c-kit, a result supported by the quantitative analysis (Fig. 4A). The thyroid gland is composed of two endocrine cell types: thyroid follicular cells and parafollicular cells (also called the C cells). Thyroid follicular cells, which make up the bulk of the gland, are derived from foregut endoderm and produce and secrete thyroid hormones T3 and T4. Parafollicular cells, on the other hand, are derived from the neuroectoderm and synthesize calcitonin. Because these thyroid parafollicular cells are mainly known for producing calcitonin, we performed IHC with an anti-calcitonin antibody to identify the C cell population. The OMP expression, detected with a specific antibody against OMP, mostly overlapped with the IHC signals from the calcitonin secreting cells (Fig. 4B). Fig. 4B illustrates the OMP immunohistochemical (IHC) expression in parafollicular cells in the interstitial regions between follicular cells, with less than 10% of the cells singularly expressing OMP (+). This colocalization of calcitonin (+) cells with OMP (+) cells indicates that the OMP in the thyroid is primarily located in the parafollicular cells.

**Fig 5:**
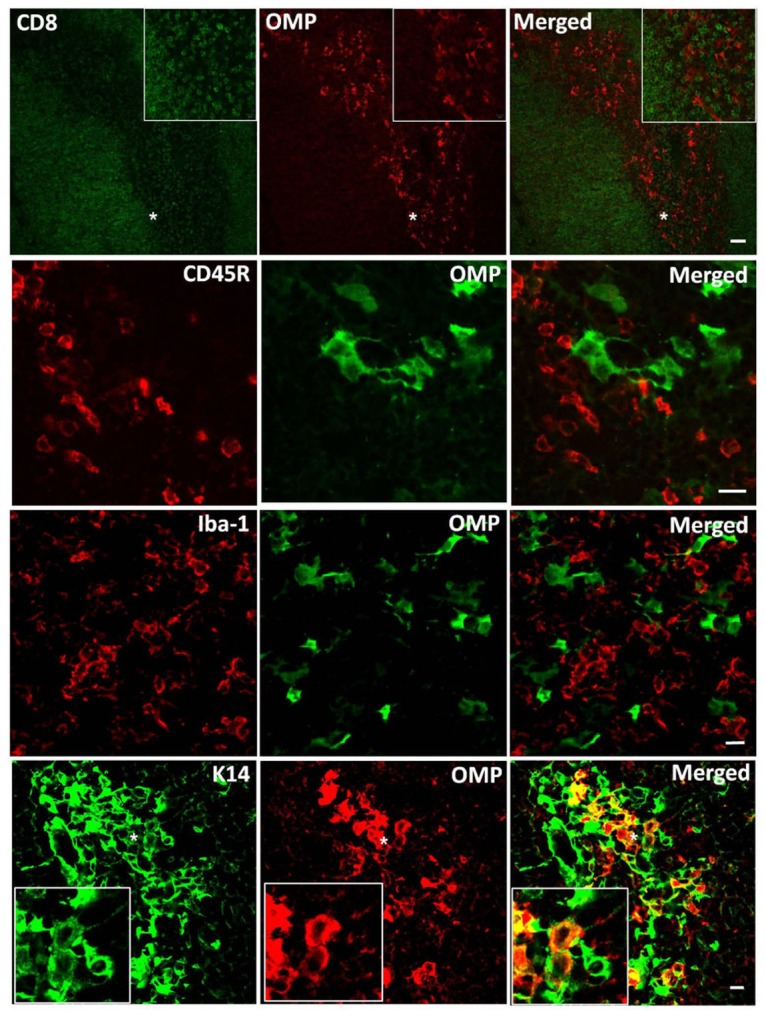
Identification of OMP (+) cells in thymus. Olfactory marker protein (OMP) expression was superimposed on each marker using double-labeling immunohistochemical assays. A representative image is shown for each antibody combination. The left panels show each marker protein (CD8 for T cells; CD45R for B cells; Iba-1 for macrophages; and keratin 14 [K14] for medullary epithelial cells). The middle panels show OMP, and the right panels are the merged images of the two individual images in the corresponding rows. OMP is co-labeled the subpopulation of K14-positive cells, but not other cell types, indicating that OMP is within thymic medullary epithelial cells (mTEC).

Finally, we examined the pattern of immunoreactivity for OMP expression in the freshly harvested thymus. A representative IHC evaluation for OMP (+) cells is illustrated in Fig. 5. Histologically, thymus has two lobes divided into many lobules, and each lobe of thymus can be divided into a central medullar and a peripheral cortex, which is surrounded by an outer capsule. The cortex and medulla play different roles in the development of T cells. Cells in the thymus can be divided into cells of hematopoietic origin for developing T cells and thymic stromal cells, which include thymic epithelial cells, thymic medullary epithelial cells (mTECs), and dendritic cells. In the present study, IHC staining of CD8 (for T cells), CD45R (for B cells), Iba-1 (for macrophages), and keratin 14 (for mTECs) revealed that the OMP (+) cells demonstrated a dense and uniform distribution within the medulla of the thymus and were entirely colocalized with the subcellular population of the keratin 14 reactive cells (Fig. 5), indicating that OMP is within the mTEC cell subpopulation.

### Identification of candidate OR genes in OMP (+) tissues

To date, there are approximately 400 OR genes in humans and more than 1,000 genes in rodents, with extensive sequence diversity within their transmembrane domains. Thus, we next assessed what types of ORs were expressed in three of the tissues (bladder, thyroid, and thymus) that showed OMP (+) immunofluorescent signals using the previously analyzed Gene Altlas2 microarray data with the bioinformatics evaluation described in Materials and Methods. To address this issue, it is mandatory to elucidate the ectopic olfactory system with potentially functional ORs. All 352 valid ORs can be ordered by their intensity values, but this is not reasonable because of the “noisy” nature of microarrays [49]. For example, if the intensity of the signal for an OR is highest in the thyroid, it is accepted that this OR is expressed in the thyroid even though the intensity of that particular OR is relatively low compared with that of other ORs in the thyroid. Thus, we utilized a ranking system based on both the tissue and the OR to address this issue. Using this analysis, the top 25 ORs manifested in each tissue were selected (S3 Table) and verified with the conventional RT-PCR using OR-specific primer sets and with sequence analysis (S3 Fig.). Prior to the RT-PCR experiments, we verified that the expression patterns of the selected ORs are significantly different from those of the ORs that were not selected. Because the OR groups were unrelated, we performed unpaired *t*-tests, specifically *t*-tests assuming unequal variances. The group size (n1) for the selected ORs was 25, and for ORs that were not selected (n2), the group size was 327. The *p*-values were sufficiently small to reject the null hypotheses (S4 Table), indicating that signals from the selected ORs were observed in the RT-PCR experiments (S3 Fig.). These results showed the expression of approximately 4–7 ORs in each tissue (S3 Fig.) and demonstrated that our approach for refining the DNA array data is effective for screening OR expression in non-olfactory systems. These RT-PCR products were sequenced, and we found that OR sequences in non-olfactory tissues are indistinguishable from those in the OE. Most of the ectopic ORs were also expressed in the OE and bulb (data not shown). These data suggest that the OR functions as a chemosensor in non-olfactory tissues as well as in OE.

To confirm the results of the conventional RT-PCR analyses and determine the relative expression of each OR, we performed quantitative real-time PCR analysis for the target ORs using olfactory bulb RNA as a positive control (Table 1). The values for the relative expression of 11 ORs were analyzed in non-olfactory tissues and olfactory bulb. Whereas olfr181 was commonly expressed in three tissues, other ORs (olfr39, olfr78, olfr288, olfr325, olfr378, olfr895, and olfr1386) were expressed in a tissue-specific manner (Table 1). A few of the ORs, such as olfr544, olfr558, and olfr1392, were commonly expressed in at least two tissues. Further study will be required to characterize the physiological responses to OR activation in non-olfactory tissues.

**Fig 6:**
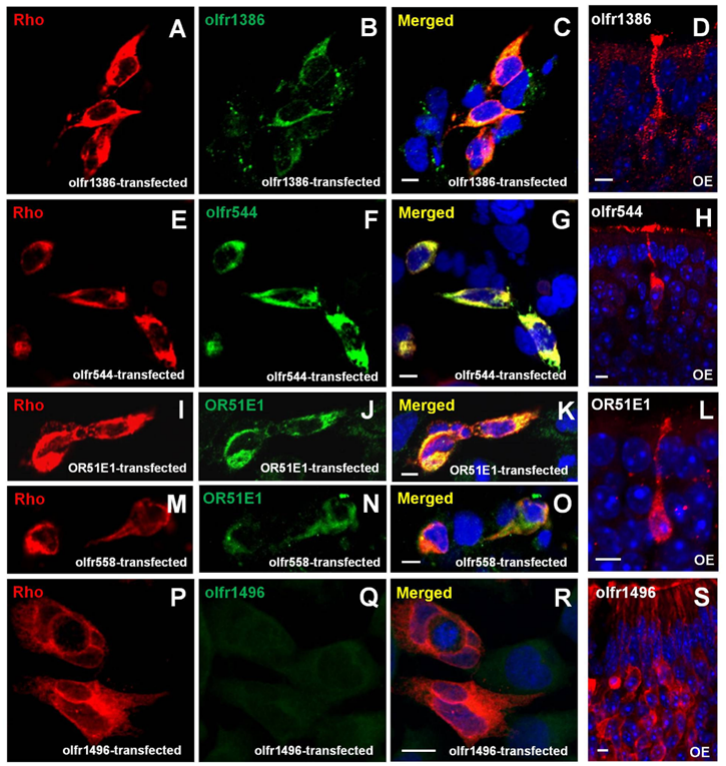
Validation of commercial antibodies against the olfactory receptor (OR). A heterologous OR expression system and the endogenous OR expression in olfactory epithelium (OE) was used to validate commercially available OR antibodies against specific ORs. HEK 293 cells were transiently transfected with cDNAs coding for several associated proteins (G_olf_, Ric8b, and RTP1S) and ORs (olfr544, olfr1386, olfr558, olfr1496, or OR51E1 [a human olfr558 ortholog]), and immunolabeled using antibodies against both the N-terminal epitope-Rho tag (A, E, I, M, and P) and each OR-specific antibody (B, F, J, N, and Q). The olfr1386 (B) and olfr544 (F) antibody detect Lucy-Flag-Rho-olfr1386- and Rho-olfr544-constructs in the transfected cells, respectively. These results are confirmed using the anti-Rho antibody (A and E; C and G for overlay). As a positive control, both antibodies recognize unique olfactory receptor neuron (ORN) (D and H) in OE. OR51E1 and anti-Rho antibody detect Rho-OR51E1 (J and I) and Rho-olfr558 (N and M) constructs in the transfected cells and ORN (L), which is expected since they share 94% amino acid identity as orthologs. The olfr1496 is detected by anti-Rho antibody (P), but not by commercial olfr1496 antibody (Q; R for overlay), which nonspecifically recognizes all ORN (S) in OE. Scale bar = 10 μm.

We next tested whether the ectopic OR is expressed in the OMP (+) cell population to provide evidence that OMP IHC is useful for cellular localization of OR-mediated events in non-olfactory tissues. However, we first examined whether commercial OR antibodies selectively recognized the appropriate OR in our immunofluorescence staining protocols using the specific OR-transfected mammalian cells and the olfactory epithelial tissue sections as positive controls (Fig. 6). The cells transiently transfected with individual OR genes tagged with a Rho epitope at the N-terminus were stained with both an anti-Rho antibody and a commercially available anti-OR antibodies. The rabbit anti-olfr1386 and anti-olfr544 antibodies specifically recognized their corresponding ORs, and these signals were co-labeled with the anti-Rho antibody (Fig. 6A-C and E-G). These antibodies also detected ORNs with distinct dendritic knobs, dendrites, and cell bodies (Fig. 6D and H). These results demonstrated the selectivity of the commercial anti-olfr1386 and anti-olfr544 antibodies. We also purchased a commercially available antibody against the human OR51E1, which is the ortholog of mouse olfr558 with an amino acid sequence identity of 93%, and tested for the cross reactivity of OR51E1 and olfr558. Both the OR51E1 and olfr558 proteins were visualized using the rabbit anti-OR51E1 antibody and detected with anti-Rho antibodies (Fig. 6I-K and M-O). Additionally, the rabbit anti-OR51E1 antibody provided a strong IHC signal in the ORNs of the mouse OE (Fig. 6L). By contrast, the anti-olfr1496 antibody did not show an immunopositive signal in olfr1496-transfected cells (Fig. 6P-R), whereas a nonspecific staining pattern of all ORNs was observed in OE (Fig. 6S). Together, these results demonstrated that anti-olfr1386, anti-olfr544, and anti-OR51E1 antibodies were selective and could be used in our experiments.

**Fig 7:**
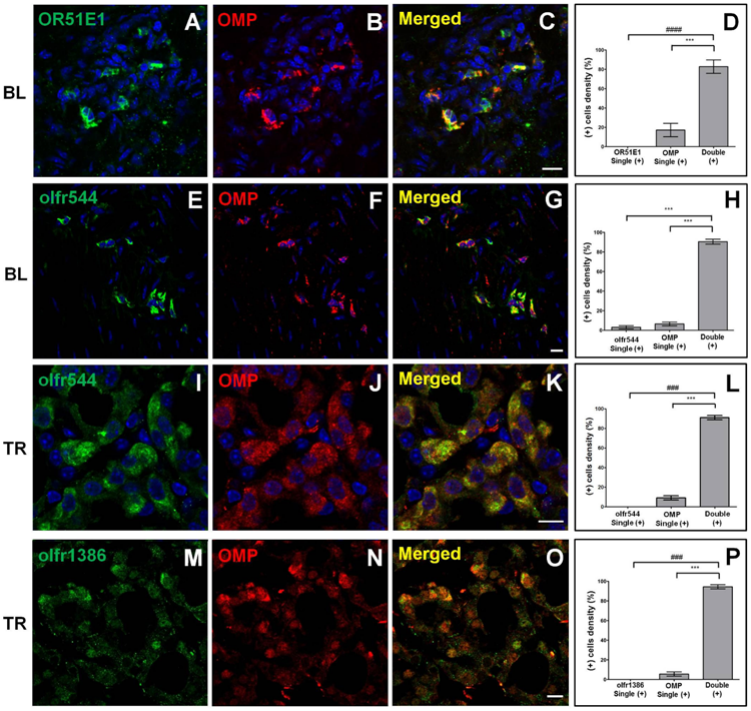
Olfactory receptors (ORs) are expressed in OMP (+) cells. Each OR is co-expressed with the olfactory marker protein (OMP) in non-olfactory tissues using co-labeling assays. A representative image is shown for each antibody combination. The left panels show each OR with Dylight 488-conjugated anti-rabbit IgG green fluorescence (A, E, I, and M). The middle panels show OMP with Cy3-conjugated anti-goat IgG red fluorescence (B, F, J, and N), and the right panels are merged images of the two individual images in the corresponding rows (C, G, K, and O). OR51E1 (mouse olfr558 ortholog; green in A) or olfr544 (green in E) is expressed with OMP (red in B and F) in the interstitial cells of Cajal (ICC) of the bladder (BL). The olfr544 (green in I) or olfr1386 (green in M) is expressed with OMP (red in J and N) in the parafollicular cells of thyroid (TR). OR (+) cells may be OMP (+) cells in each cell type of mouse tissue as evidenced by the nearly complete overlap of OR and OMP expression. Nuclei are counterstained with DAPI (blue fluorescence), except the image for olfr1386 in thyroid. OR51E1 (olfr558)/OMP (D), olfr544/OMP (H), olfr544/OMP (L), and olfr1386/OMP (P) colocalization is supported by quantitative analysis. ***, *P* < 0.0001 using unpaired *t*-test; ###, *P* = 0.0001, and ####, *P* < 0.0001 using the Mann—Whitney *U* test OMP + OR51E1 (olfr558), olfr544, or olfr1386 versus each OR or OMP alone. Results are the means ± SEM of three or four independent areas (n = 3–5 mice). Scale bar: 10 μm.

Previous results in the present study confirmed that several ORs were expressed in the OMP (+) tissues. However, evidence that ORs were co-expressed with the OMP in the same cell of each tissue was lacking. To address this issue, we tested whether ORs were co-expressed with the OMP using the validated anti-olfr544, anti-olfr1386, and anti-OR51E1 antibodies. Surprisingly, all ORs tested in this study were expressed in the OMP (+) cells (Fig. 7). We observed few OMP (+) cells without colocalization of an investigated OR. In the bladder, greater than 80% of the OMP signal was co-expressed with OR51E1 (olfr558), and less than 20% of the OMP signal was expressed alone (Fig. 7A-D). Greater than 95% of the immunoreactivity for olfr544 and OMP was observed in the ICC of the bladder (Fig. 7E-H). For the thyroid, OMP was expressed alone in less than 5% of the OMP (+) cells, and neither olfr544 nor olfr1386 was expressed in the absence of the OMP. Thus, all olfr544- or olfr1386-positive cells were also OMP (+) (Fig. 7I-L and M-P). These results indicate that OMP IHC analysis is a useful tool for determining the cellular location of expressed ectopic ORs.

## Discussion

In 1991, Buck and Axel discovered both the family of transmembrane proteins that were believed to be odor receptors and some of the genes for these proteins, a seminal breakthrough in the knowledge of the olfactory system [50]. Olfactory sensing is mediated by the ORN. ORNs, primary neurons of the olfactory system, are distinctive because of their short lifespan and continual replacement from neural progenitor cells [51]. In addition, ORs have been increasingly identified in non-olfactory tissues [28,29] and may have both olfactory and non-olfactory functions. Accompanying the ORs, despite the uncertainty of its physiological function, OMP is highly expressed in primary ORNs [21,52]. It is also found in restricted areas of the CNS such as the hypothalamus in several vertebrate species [53,54]. The molecular genetics and structure of the OMP are now well-established [55,56]. This protein has a highly selective expression pattern and is widely accepted as a marker of the mature ORNs in the main OE, vomeronasal epithelium, septal organ, and Grunenberg ganglion [21,52,57,58]. The OMP plays a pivotal role in one of the earliest steps of olfactory transduction in the ORNs and is a participant in the overall OR-associated signal transduction cascade [24,27,59]. Since the initial description by Margolis more than 40 years ago [60], The OMP and its promoter have served as critical reagents for the study of the vertebrate olfactory system [61–63].

The basics of odor perception and signaling from olfactory cilia to the brain are well-documented [64]. However, although the knowledge of the expression and functional roles of the ORs in non-olfactory tissues has been extended significantly [28], the identification of additional expression profiles and physiological functions of the ORs in non-olfactory tissues remains unknown, primarily because of the low expression levels of OR genes or proteins in non-olfactory tissues coupled with the absence of specific antibodies due to the lack of sequence identities of specific ORs. In this study, to overcome the limitations of using ORs as an experimental target, we used the OMP to screen for potential OR-mediated sensing systems in non-olfactory tissues. The presence of OMP in several non-olfactory tissues was confirmed by the combined results of conventional RT-PCR, double immunoassays, and double-labeling IHC assays. OMP was considered virtually restricted to the olfactory system, with the exception of several small sets of neurons in the central nervous system and the hypothalamus [53,54]. However, unexpectedly, our primary screening results using conventional RT-PCR demonstrated the existence of OMP transcripts in several non-olfactory tissues, and our subsequent double immunoassays showed that OMP protein was also expressed in some non-olfactory tissues (Fig. 1). We are the first in the field of non-olfactory signaling systems to show that OMP is expressed in non-neuronal tissues, suggesting that it may play a more general role in chemosensing in addition to its role in the olfactory system. To determine the localization of the OMP expression in these tissues, we used an IHC approach using goat anti-OMP serum [57]. Out of the 13 non-olfactory tissues examined, OMP protein expression was clearly detectable in five tissues, including bladder, thyroid, thymus, heart, and testis, but remained undetectable in the other tissues. This observation expanded the localization of OMP (+) cells beyond the OE to include these five tissues. Furthermore, OMP was expressed in cytologically specified areas, namely, the Leydig cells of the testis (data not shown), parafollicular cells of the thyroid, and ICC of the bladder (Fig. 4). The bladder ICC respond to neurogenic stimulation, giving a hint for their functional innervation and indicating that bladder ICC subpopulations are under direct control of the complex innervation that governs normal bladder function. In addition, morphological and ultrastructural evidence has shown the physical relationship between ICC and intramural nerves [65]. Parafollicular cells of the thyroid are neural crest cell derivatives and are mainly known for secreting calcitonin, a plasma calcium-decreasing hormone. Calcitonin-producing parafollicular cells co-express neuroendocrine peptides, indicating the involvement of neuronal signaling events [66]. These results were confirmed with antibodies against OMP and other marker proteins such as Glial fibrillary acidic protein (GFAP), calcitonin, and c-kit [46,66,67]. The OMP (+) cell types of these three non-chemosensory tissues (testis, bladder, and thyroid) are considered to be within the neuroendocrine system, which is made up of the nervous and endocrine systems and interposition properties, like interstitial cells. However, unlike these three non-olfactory tissues, in the thymus, the OMP (+) cells are medullary thymic epithelial cells (mTEC), as confirmed by keratin 14 expression, and thus are not part of the neuroendocrine system. This result may provide new insights for mTEC studies examining neuronal innervation.

After identifying the OMP (+) cell populations, we determined the co-expression of OMP with other components of the OR-mediated signal transduction cascade because deletion of OMP clearly influences OR-mediated signal transduction in the olfactory system [23,26]. In previous reports, the function of ectopic ORs has been suggested by their association with ACIII and G_olf_ in skeletal muscle and kidney [32,35]. Our results also provide evidence for the co-expression of OMP, ACIII, and G_olf_ in bladder and thymus, suggesting that these tissues may use the canonical OR-mediated downstream signaling molecules. This observation along with previous studies expanded our knowledge of the presence of OMP (+) cells beyond OE to several non-olfactory tissues. In addition, canonical OR-mediated signal transduction molecules, such as OMP, ACIII, and G_olf_, were co-expressed in these tissues. Based on these results, we speculate that OMP is involved in the OR-mediated signal transduction cascades with canonical olfactory signaling components between the nervous and endocrine systems.

In identifying which cells in each of these tissues express ORs, the large size of the OR gene family can be an obstacle. Therefore, we chose an alternate strategy and screened each tissue using the IHC detection of an OMP antibody as a probe to identify cell populations potentially using the OR-mediated signaling pathway. However, no direct evidence indicated that ORs were co-expressed with OMP in the same cell of each tissue. To find OR expression in OMP (+) tissues, we initially selected 25 ORs in each tissue using the refined Gene Atlas2 microarray data and then performed conventional RT-PCR and quantitative real-time PCR to confirm OR expression in the OMP (+) tissues. These results showed that several OR mRNAs were expressed in the OMP (+) tissues. We next determined whether the proteins for the ORs selected from this result were expressed in the same cells with OMP. However, this required antibodies selective against specific ORs. Therefore, we validated the specificity of a few high quality commercial OR antibodies against the ORs selected in the previous quantitative real-time PCR experiments and used these validated antibodies in experiments designed to co-label with the OMP. The results showed that olfr558 (a human OR51E1 ortholog) and olfr544 were each co-expressed with the OMP in the interstitial cells of the bladder and that olfr1386 and olfr544 were co-expressed with OMP in the parafollicular cells of the thyroid. These results suggest that using an IHC approach with OMP may be a useful and effective alternate tool to identify the cellular location of ectopic OR-mediated sensing, instead of using ORs themselves as a target. Thus, ectopic OMP expression is a valid option to bypass the limitations involved in directly investigating ectopic OR expression and a better approach in systematically searching tissues for an operating ectopic OR-mediated signaling pathway.

In conclusion, the present study demonstrated that the transcript and protein of OMP were widely expressed in non-olfactory tissues and that OMP and ORs, as well as G_olf_ and ACIII, were co-expressed in specific subpopulations of cells in the bladder and thyroid. Our data suggest that OR expression can be inferred from OMP expression and that the ectopic OR-mediated signaling pathway is present in several non-olfactory tissues. In the future, it will be important to characterize the functional roles of ectopic ORs to determine the ligands that activate these signaling pathways and the physiological functions they mediate.

## Supporting Information

S1 Fig Expression of olfactory marker protein (OMP) mRNA in various mouse tissues. **(A)** The mRNA of OMP is expressed in most mouse tissues at different levels. As expected, OMP mRNA is most abundant in olfactory epithelium (OE) as a positive control. OMP is detected in various non-olfactory tissues. **(B)** Quantification of OMP mRNA expression levels in mouse tissues. The ratios (%) of each band relative to the control (β-actin) are designated as the relative expression level of each mRNA. Experiments were performed three times independently, and all data shown are from representative experiments. RT (-) is a negative control without reverse transcriptase, and β-actin is a loading control. M indicates a molecular weight marker. LV, liver; SM, skeletal muscle; BL, bladder; PC, pancreas; ST, stomach; DD, duodenum; TT, testis; SP, spleen; HT, heart; TM, thymus; KD, kidney; TR, thyroid; LG, lung.
(TIF)

S2 Fig Olfactory marker protein (OMP)-specific (asterisks) and autofluorescence (arrows) signals differ in testis. Characteristic autofluorescence signals are detected in interstitial cells of testis. Arrows indicate interstitial cells expressing autofluorescence as red signals. The asterisks show specific OMP signals that clearly differ from the autofluorescence. NGS (normal goat serum) was used as a negative control in this experiment.
(TIF)

S3 Fig Validation of olfactory receptor (OR) expression identified from the refined microarray data using RT-PCR. OR expression was evaluated with cDNA samples of bladder, thyroid, and thymus in the presence (+) and absence (-) of reverse transcriptase to demonstrate a lack of genomic DNA contamination using RT-PCR analysis. Only olfr181 is commonly expressed in all three tissues, whereas other ORs, including olfr39, 78, 288, 325, 378, 895, and 1386, are only expressed in one of the investigated tissues. Identification of the ORs was confirmed by sequencing.
(TIF)

S1 Table Primers used in this study. (DOCX)

S2 Table Primary antibodies used in this study. (DOC)

S3 Table Top 25 olfactory receptors defined using the refined microarray analysis. (DOCX)

S4 Table p-Values from unpaired *t*-tests for three tissues (n1 = 25 and n2 = 327). (DOCX)
