# Supplementary figures and images for: Olfactory Marker Protein Expression Is an Indicator of Olfactory Receptor-Associated Events in Non-Olfactory Tissues
Source: PLoS One. 2015 Jan 30;10(1):e0116097. doi: 10.1371/journal.pone.0116097 (PMC4311928; doi:10.1371/journal.pone.0116097)

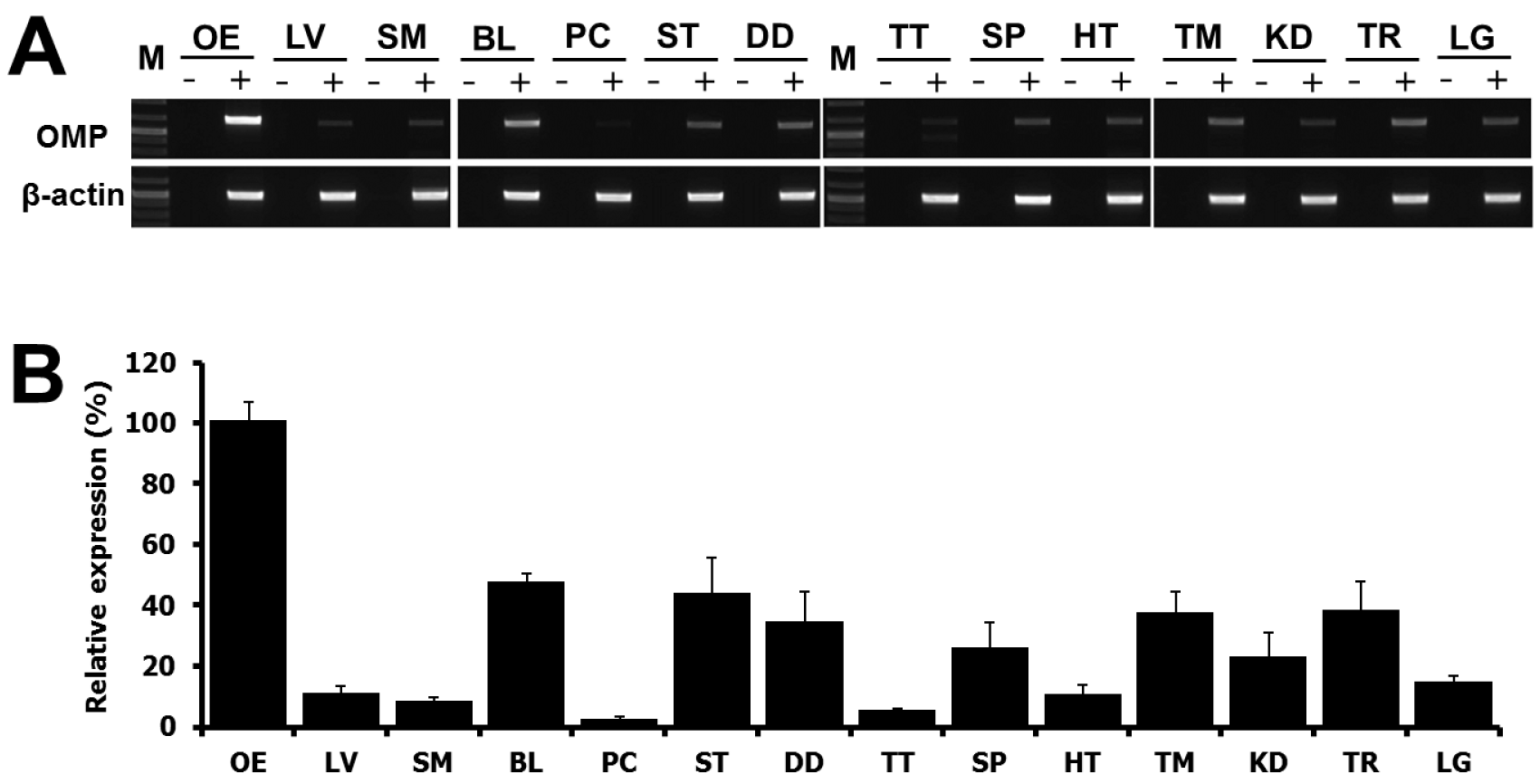

Supplement: S1 Fig — (A) The mRNA of OMP is expressed in most mouse tissues at different levels. As expected, OMP mRNA is most abundant in olfactory epithelium (OE) as a positive control. OMP is detected in various non-olfactory tissues. (B) Quantification of OMP mRNA expression levels in mouse tissues. The ratios (%) of each band relative to the control (β-actin) are designated as the relative expression level of each mRNA. Experiments were performed three times independently, and all data shown are from representative experiments. RT (-) is a negative control without reverse transcriptase, and β-actin is a loading control. M indicates a molecular weight marker. LV, liver; SM, skeletal muscle; BL, bladder; PC, pancreas; ST, stomach; DD, duodenum; TT, testis; SP, spleen; HT, heart; TM, thymus; KD, kidney; TR, thyroid; LG, lung. (TIF) [file pone.0116097.s001.tif]

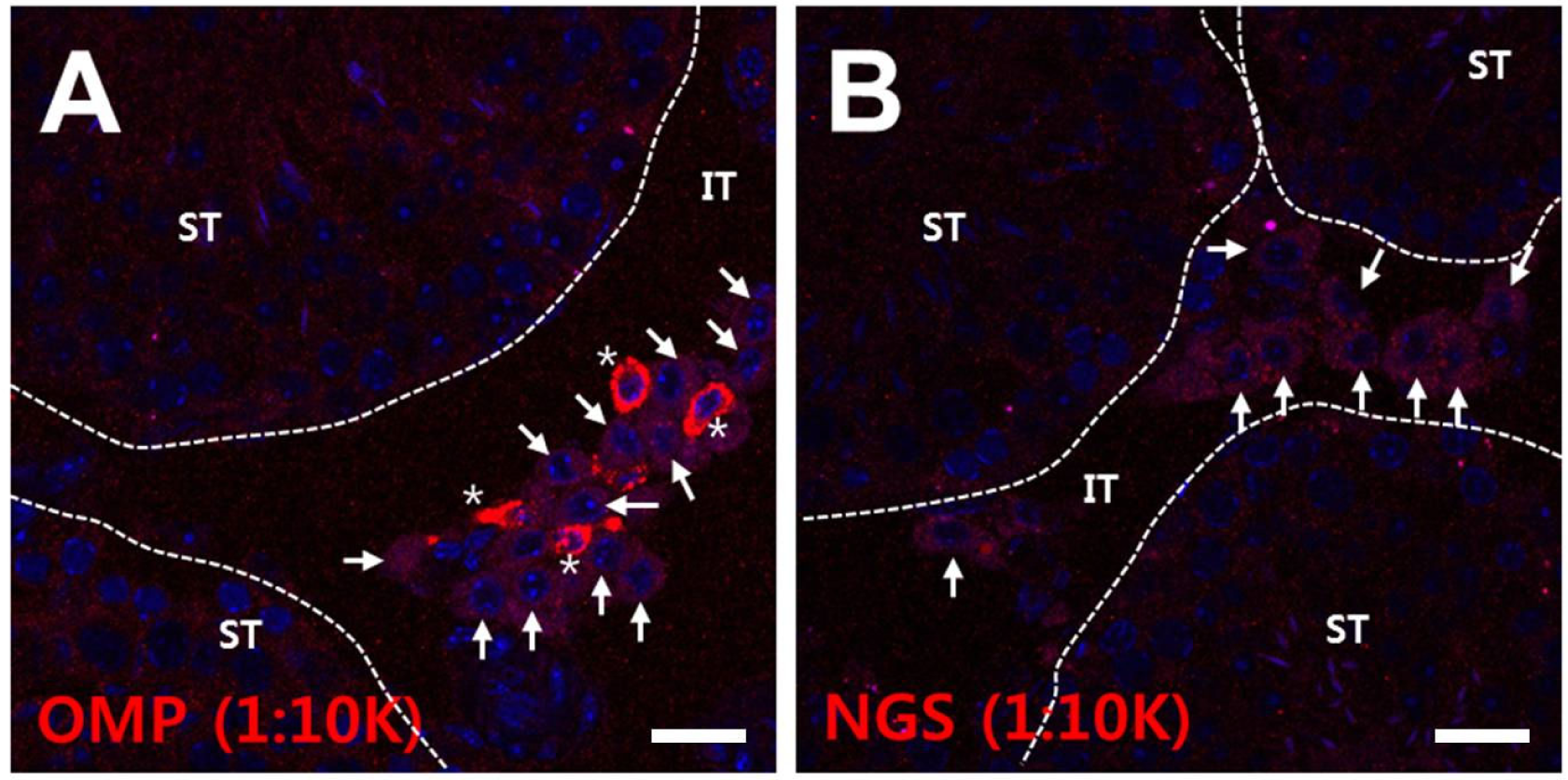

Supplement: S2 Fig — Characteristic autofluorescence signals are detected in interstitial cells of testis. Arrows indicate interstitial cells expressing autofluorescence as red signals. The asterisks show specific OMP signals that clearly differ from the autofluorescence. NGS (normal goat serum) was used as a negative control in this experiment. (TIF) [file pone.0116097.s002.tif]

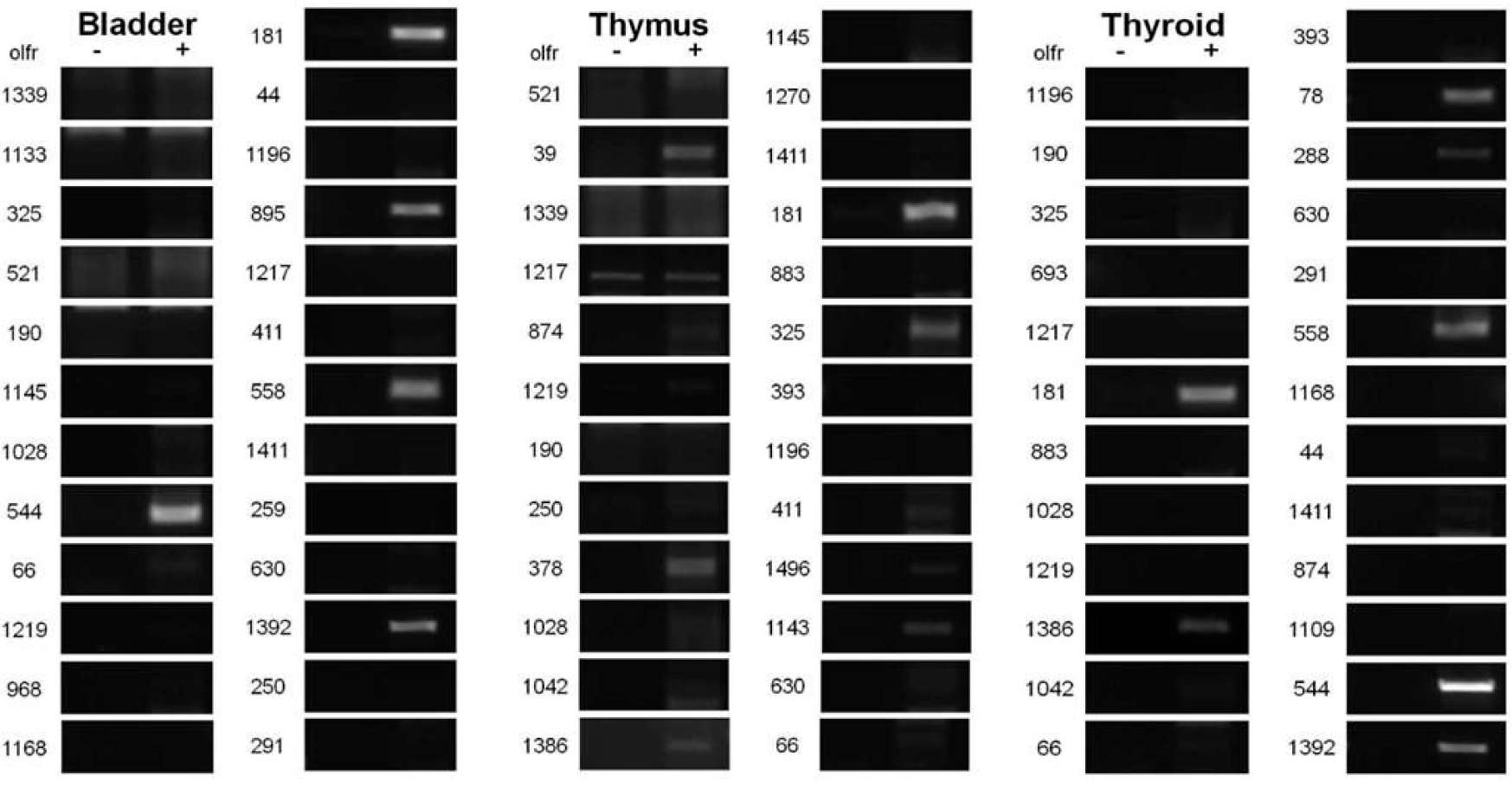

Supplement: S3 Fig — OR expression was evaluated with cDNA samples of bladder, thyroid, and thymus in the presence (+) and absence (-) of reverse transcriptase to demonstrate a lack of genomic DNA contamination using RT-PCR analysis. Only olfr181 is commonly expressed in all three tissues, whereas other ORs, including olfr39, 78, 288, 325, 378, 895, and 1386, are only expressed in one of the investigated tissues. Identification of the ORs was confirmed by sequencing. (TIF) [file pone.0116097.s003.tif]
