# Supplementary material for: Olfactory Marker Protein Expression Is an Indicator of Olfactory Receptor-Associated Events in Non-Olfactory Tissues
Source: PLoS One. 2015 Jan 30;10(1):e0116097. doi: 10.1371/journal.pone.0116097 (PMC4311928; doi:10.1371/journal.pone.0116097)
Supplement: S1 Table — (DOCX) [file pone.0116097.s004.docx]

**Table S1. Primers used in this study**

| **olfr** | **Sequence** | | **Size (bp)** | **olfr** | **Sequence** | | **Size (bp)** |
| --- | --- | --- | --- | --- | --- | --- | --- |
| 39 | F | TCGCAGATTGTCTCCTCCTT | 248 | 291 | qF | CCCACTGATGTAATTTGTTGCTGG | 270 |
|  | R | AGAGCCCCCTTCACATCTTT |  |  | qR | GATAGCGCACTTCAGGGAGG |  |
|  | qF | CTCCTACATGCAGTGCCTCA | 90 | 325 | F | TGCTCAGACACCTGGCTCTA | 454 |
|  | qR | CGGTCAAAGGCCATTACAGT |  |  | R | ATCAGCATGACACCTGGAAA |  |
| 44 | F | CTGCCCCTCTTCTTCCTTTT | 225 |  | qF | ACCCCTCTGTTGAACCCACT | 171 |
|  | R | AGTCATGCATCCAGGGTAGG |  |  | qR | GCATGACACCTGGAAAAACAGA |  |
| 66 | F | TCTGGGCTTGGAAATGATTC | 162 | 378 | F | CCAGAGCACCAGCAACTGTA | 294 |
|  | R | AGGTCCATACTTGCCAGCAC |  |  | R | CATGGCAACAAGGAGGAAGT |  |
| 78 | F | CGCTGCTGTCCTCAACAATA | 107 |  | qF | ACTCTGCTTAAAGTGGCTTGTTCT | 101 |
|  | R | AAGCCAGTCGCTTGATCAGT |  |  | qR | ACAATGATGAGAAGGAAAGGGAGT |  |
| 181 | F | GGAAATCTGGCTCTGATGGA | 208 | 393 | F | CTGTCACAATGCCCAAAATG | 203 |
|  | R | GGTACTGCAGAGGGTTGCAT |  |  | R | AGTACAGAGCTTGGGGCTCA |  |
|  | qF | CACAGATCAAGCATGATGAAGG | 113 |  | qF | GCTGCTACTGTGGATACTGACGA | 130 |
|  | qR | ATGGCAGAGAACAGCAGGAA |  |  | qR | GGACAGCTTTAGGACAGCAAAT |  |
| 190 | F | TGTGACATCGTTCCATTGCT | 177 | 411 | F | GGGCTTGGTAGAGACAGCAG | 158 |
|  | R | AGCCTTCTTCATGCTTTGGA |  |  | R | TGGACAGATTCCCCAGAAAG |  |
|  | qF | AAGTCCTCACCGTTGTGATTG | 92 | 521 | F | AGACACCACTCCCCAGACAC | 290 |
|  | qR | AGCCTTCTTCATGCTTTGGA |  |  | R | GGTTGAGAACAGGGGTGAGA |  |
| 250 | F | TTTACCCCAAAAATGCTGATGG | 231 | 544 | F | GCATCTACTTCCTTATTGTCTTT | 678 |
|  | R | GACACCCATCAAGTGTGCAG |  |  | R | TTGCTGAGTCCTTTGGAACAGAGC |  |
| 259 | F | ACCCTAATTGGCAGGTTCCTC | 235 |  | qF | GGACATCTCGCTGAATAAGACG | 59 |
|  | R | ATACAGGCAAACAGCCTCGT |  |  | qR | CCAGGACTCGGTTGAAGATG |  |
| 288 | F | CATCTGCCACCCACTGAACT | 598 | 558 | F | TGGGGGAAAAGACACACAG | 170 |
|  | R | ACTTCTCCAGACACCCTCCTT |  |  | R | GCCAGCCAAAACTGAACCT |  |
|  | qF | CATCTACAGCTTCCGCAACA | 188 | 630 | F | CCTCAAACTCCAGCACCATT | 179 |
|  | qR | TGGCAAGGCTCCTTTTTAGA |  |  | R | ATACATGGGCTCGTGGAGTC |  |

| 874 | F | GCACCTTCATAAATGAGCTCGTAA | 295 | 1196 | F | CCAACTCTGGGATGGTGCTTTT | 328 |
| --- | --- | --- | --- | --- | --- | --- | --- |
|  | R | ATCAGAGGGTTCAACATGGGC |  |  | R | TCAACTCTGAGTGACAGCCGA |  |
| 883 | F | TATCCTGCTGGGCTTGACAC | 446 | 1217 | F | TTTGCAGCTGCCTTTATGTG | 216 |
|  | R | TACCAGTGTGTGCAATGGCTT |  |  | R | TTACGTCGCCCTTCAGAACT |  |
| 895 | F | GCTCTCCTGCAGTAGCACCT | 250 | 1219 | F | TTGGGGGCAACATGATTATT | 221 |
|  | R | TCCCACAGTCTCAGCAGATG |  |  | R | TTCTGCCCCAGTGAAGAAGT |  |
|  | qF | GGCTTGTTAGTGCCTTATTCCATAC | 154 | 1270 | F | TGCAGCCATTATTCAAGCTG | 218 |
|  | qR | ACCACAGCAGAGCTCACAAG |  |  | R | GGGTCCAAAGAACAAGACGA |  |
| 968 | F | TCCCCACTCCTGGAACTCTC | 473 | 1339 | F | CATCCTTCTGGGTTTCTCCA | 175 |
|  | R | TTGTGGTGGCCATACTTAGCA |  |  | R | ATCCAGCATGGAAAGGACAC |  |
|  | qF | CCCATGCTGAATCCTTTGAT | 169 | 1386 | F | CCTGGTTCAGGAAATGCTGT | 153 |
|  | qR | TGTGGTGGCCATACTTAGCA |  |  | R | AGCTGAGGCCAATCTGAAAA |  |
| 1028 | F | TGGATTCCCGCCTTCACAAG | 486 |  | qF | AGGGAGAGGCACAGACTAAGA | 104 |
|  | R | TTGTAAGGGAGCTGGAAAGGT |  |  | qR | TGGTCGGTAGAACAGGGTAGA |  |
| 1042 | F | CATGCCTAGGGAGCTCTGTC | 208 | 1392 | F | ATGAACATCATGCACCCCGT | 551 |
|  | R | GCCAGCAATGATTAGCAACA |  |  | R | GCATTTCCCTCCTACCCTGT |  |
| 1109 | F | TGCAACCACAGAAGTCATCC | 198 |  | qF | CCCCATGTACTACTTCCTCTGC | 147 |
|  | R | ACGTTGGGACCACAGAAGTC |  |  | qR | GCAAGAGTTATGAAGAGCTGAGAC |  |
| 1133 | F | TGGCTGTGTGGTAGGGTGTA | 215 | 1411 | F | CAGGAATGGAAGCCTCTCAG | 552 |
|  | R | AGGCCATTTTGTCTGTGTCC |  |  | R | GGGGGAACGTCACAGTAGAA |  |
| 1143 | F | TGGGTTCATTGAATTGAGCA | 187 | 1496 | F | CCAGGCTGAGAAGCAAAAAC | 234 |
|  | R | AGAAGAACTTGGCCGGAAAT |  |  | R | CAGCCCTCATAGGAAATGGA |  |
| 1145 | F | CTCCTGGCTAAGTGGCATTC | 185 | OMP^a^ | F | AAGCTGCAGTTCGATCACTG | 682 |
|  | R | ATGCAGCCACAACCATAACA |  |  | R | TGTTCCTGTCCAGTCTCAGTCT |  |
| 1168 | F | TGGCCTATGACAGGTTTGTG | 282 | β-actin | F | ATATCGCTGCGCTGGTCGTG | 517 |
|  | R | GAAGGCTACTACCCTCACTTA |  |  | R | AGGATGGCGTGAGGGAGAGC |  |

^a^ Includes primers used in [1].

F, forward primer for the conventional RT-PCR; R, reverse primer for the conventional RT- PCR; qF, forward primer for qPCR; qR, reverse primer for qPCR.

**Reference**

1. Buiakova OI, Baker H, Scott JW, Farbman A, Kream R, et al. (1996) Olfactory marker protein (OMP) gene deletion causes altered physiological activity of olfactory sensory neurons*.* Proc Natl Acad Sci U.S.A. 93: 9858-9863.
