# Supplementary material for: Olfactory Marker Protein Expression Is an Indicator of Olfactory Receptor-Associated Events in Non-Olfactory Tissues
Source: PLoS One. 2015 Jan 30;10(1):e0116097. doi: 10.1371/journal.pone.0116097 (PMC4311928; doi:10.1371/journal.pone.0116097)
Supplement: S2 Table — (DOC) [file pone.0116097.s005.doc]

### Table S2. Primary antibodies used in this study

| **Antibody** | **Immunogen** | **Source or reference** | **Species** | **Dilution** |
| --- | --- | --- | --- | --- |
| OMP | Rat OMP | [1] | Goat polyclonal | 1:5,000 |
| OMP | Rat OMP | [1] | Rabbit polyclonal | 1:20,000 |
| ACIII | Mouse C-terminal  C-20 peptide | Santa Cruz Biotechnology  (cat. no. sc-588) | Rabbit affinity-purified  polyclonal | 1:500 |
| G_olf_ | Rat divergent domain peptide | Santa Cruz Biotechnology  (cat. no. sc-385) | Rabbit affinity-purified  polyclonal | 1:200 |
| c-kit | Human C-terminal  C-19 peptide | Santa Cruz Biotechnology  (cat. no. sc-168) | Rabbit affinity-purified  polyclonal | 1:200 |
| Calcitonin | Synthetic human calcitonin, aa 1-32 | DakoCytomation  (Code-Nr. A 0576) | Purified immunoglobulin fraction of rabbit antiserum | 1:100 |
| CD45R | Exon A-restricted isoform of mouse CD45 | eBioscience  (cat. no. 12-0452) | Rat monoclonal | 1:200 |
| Iba-1 | Iba-1 C-terminal peptide | Wako  (cat. no. 019-19741) | Rabbit affinity-purified polyclonal | 1:500 |
| K14 | Mouse keratin 14 C-terminal peptide | Covance  (cat. no. PRB-155P) | Rabbit affinity-purified polyclonal | 1:200 |
| CD8 | Mouse thymus/spleen cells | BD Pharmingen  (cat. no. 550281) | Mouse affinity-purified monoclonal | 1:200 |
| olfr1386 | Mouse olfr1386 C-terminal aa 259-308 | Abcam  (cat. no. ab128276) | Rabbit affinity-purified polyclonal | 1:200 |
| olfr544 | Mouse olfr544 peptide | Abcam  (cat. no. ab65528) | Rabbit polyclonal | 1:200 |
| OR51E1 | Human OR51E1 C-terminal peptide | LSBio  (cat. no. LS-A1854) | Rabbit affinity-purified polyclonal | 1:200 |
| olfr1496 | Mouse olfr1496 peptide | Abcam  (cat. no. ab69187) | Rabbit polyclonal whole antiserum | 1:200 |
| Rhodopsin | Rat rhodopsin N-terminal peptide | Millipore  (cat. no. MABN15) | Mouse affinity-purified monoclonal | 1:1,000 |
|  |  |  |  |  |

**Reference**

1. Margolis FL (1972) A brain protein unique to the olfactory bulb. Proc Natl Acad Sci U S A. 69: 1221-1224.
