# Supplementary material for: Olfactory Marker Protein Expression Is an Indicator of Olfactory Receptor-Associated Events in Non-Olfactory Tissues
Source: PLoS One. 2015 Jan 30;10(1):e0116097. doi: 10.1371/journal.pone.0116097 (PMC4311928; doi:10.1371/journal.pone.0116097)
Supplement: S3 Table — (DOCX) [file pone.0116097.s006.docx]

**Table S3. Top 25 olfactory receptors defined using the refined microarray analysis**

| **Rank** | **Bladder** | **Thymus** | **Thyroid** |
| --- | --- | --- | --- |
| 1 | Olfr1339 | Olfr521 | Olfr1196 |
| 2 | Olfr1133 | Olfr39 | Olfr190 |
| 3 | Olfr325 | Olfr1339 | Olfr325 |
| 4 | Olfr521 | Olfr1217 | Olfr693 |
| 5 | Olfr190 | Olfr874 | Olfr1217 |
| 6 | Olfr1145 | Olfr1219 | Olfr181 |
| 7 | Olfr1028 | Olfr190 | Olfr883 |
| 8 | Olfr544 | Olfr250 | Olfr1028 |
| 9 | Olfr66 | Olfr378 | Olfr1219 |
| 10 | Olfr1219 | Olfr1028 | Olfr1386 |
| 11 | Olfr968 | Olfr1042 | Olfr1042 |
| 12 | Olfr1168 | Olfr1386 | Olfr66 |
| 13 | Olfr181 | Olfr1145 | Olfr393 |
| 14 | Olfr44 | Olfr1270 | Olfr78 |
| 15 | Olfr1196 | Olfr1411 | Olfr288 |
| 16 | Olfr895 | Olfr181 | Olfr630 |
| 17 | Olfr1217 | Olfr883 | Olfr291 |
| 18 | Olfr411 | Olfr325 | Olfr558 |
| 19 | Olfr558 | Olfr393 | Olfr1168 |
| 20 | Olfr1411 | Olfr1196 | Olfr44 |
| 21 | Olfr259 | Olfr411 | Olfr1411 |
| 22 | Olfr630 | Olfr1496 | Olfr874 |
| 23 | Olfr1392 | Olfr1143 | Olfr1109 |
| 24 | Olfr250 | Olfr630 | Olfr544 |
| 25 | Olfr291 | Olfr66 | Olfr1392 |
