# Supplementary material for: Olfactory Marker Protein Expression Is an Indicator of Olfactory Receptor-Associated Events in Non-Olfactory Tissues
Source: PLoS One. 2015 Jan 30;10(1):e0116097. doi: 10.1371/journal.pone.0116097 (PMC4311928; doi:10.1371/journal.pone.0116097)
Supplement: S4 Table — (DOCX) [file pone.0116097.s007.docx]

**Table S4. *p*-Values from unpaired *t*-tests for three tissues (n1 = 25 and n2 = 327).**

| **Tissue** | ***p*-value** |
| --- | --- |
| Bladder | 5.6873E-04 |
| Thymus | 2.0151E-05 |
| Thyroid | 1.7437E-05 |
